# Enhancing care in alcohol-associated liver disease through peer support for alcohol use disorder

**DOI:** 10.1097/HC9.0000000000000843

**Published:** 2026-01-09

**Authors:** Jenn Jones, Lorrinda Gray-Davis, Lorenzo Leggio, Andrea DiMartini, Suthat Liangpunsakul

**Affiliations:** 1 Sober Livers, Fatty Liver Foundation, Denver, Colorado, USA; 2 Transplant Recipient International, Temple, Texas, USA; 3 Clinical Psychoneuroendocrinology and Neuropsychopharmacology Section, Translational Addiction Medicine Branch, National Institute on Drug Abuse Intramural Research Program and National Institute on Alcohol Abuse and Alcoholism Division of Intramural Clinical and Biological Research, National Institutes of Health, Baltimore, Maryland, USA; 4 Department of Behavioral and Social Sciences, Center for Alcohol and Addiction Studies, Brown University, Providence, Rhode Island, USA; 5 Department of Medicine, Division of Addiction Medicine, School of Medicine, Johns Hopkins University, Baltimore, Maryland, USA; 6 Department of Neuroscience, Georgetown University Medical Center, Washington, District of Columbia, USA; 7 Starzl Transplantation Institute, University of Pittsburgh School of Medicine, Pittsburgh, Pennsylvania, USA; 8 Department of Surgery, University of Pittsburgh School of Medicine, Pittsburgh, Pennsylvania, USA; 9 Department of Psychiatry, University of Pittsburgh School of Medicine, Pittsburgh, Pennsylvania, USA; 10 Division of Gastroenterology and Hepatology, Department of Medicine, Indiana University School of Medicine, Indianapolis, Indiana, USA; 11 Department of Biochemistry and Molecular Biology, Indiana University School of Medicine, Indianapolis, Indiana, USA; 12 Roudebush Veterans Administration Medical Center, Indianapolis, Indiana, USA

**Keywords:** alcohol use disorder, alcohol-associated liver disease, care improvement, peer support

## Abstract

Alcohol use disorder (AUD) and alcohol-associated liver disease (ALD) are interconnected conditions that contribute significantly to global morbidity and mortality. Despite advances in medical management, care for individuals with AUD and ALD remains suboptimal due to persistent gaps in psychosocial support, stigma, and limited integration between behavioral health care including AUD treatment services and hepatology. Peer support, emotional, informational, and practical assistance provided by individuals with lived experience, has emerged as a promising, though underutilized, strategy to address these challenges. This review examines the evolving role of peer and patient support programs, including community-based groups such as Alcoholics Anonymous and SMART Recovery, structured interventions for transplant candidates, and the integration of peer navigators within medical settings. Evidence suggests that peer support fosters recovery by enhancing engagement, reducing isolation, and promoting self-efficacy. Technological innovations, including virtual platforms and mobile apps, are extending the reach of peer support, particularly in rural or underserved populations. In addition, culturally tailored and demographically specific models are increasingly being adopted to address the diverse needs of patients. However, several challenges persist, including variability in peer training, inconsistent oversight, and limited research specific to ALD populations. To maximize the impact of peer support, future efforts must focus on integrating these programs into clinical care, ensuring sustainable funding, and evaluating outcomes using standardized metrics. Peer support represents a critical opportunity to enhance the recovery experience for individuals with AUD and ALD by addressing the emotional and social dimensions of care often overlooked in traditional medical settings.

Alcohol use disorder (AUD) is a prevalent and debilitating condition, affecting millions of individuals globally and contributing significantly to the burden of disease.^1^ Among its most severe consequences is alcohol-associated liver disease (ALD), which encompasses a spectrum of histopathological changes ranging from steatosis to advanced conditions such as alcohol-associated hepatitis, cirrhosis, and hepatocellular carcinoma.^1^ ALD is a leading cause of liver-related mortality, accounting for nearly half of all liver disease deaths globally,^2^^,^^3^ and is the leading indication for liver transplantation in the United States and Europe.^4^ Despite advancements in understanding the pathophysiology of AUD and ALD, patient care remains challenging. High rates of recurrence, stigma, limited access to specialized care, and inadequate integration of psychosocial support with medical management create significant barriers to achieving optimal outcomes.^5^^–^^7^ In fact, unlike for other areas of hepatology (eg, hepatitis B and C management that saw the integration of hepatology and infectious disease expertise), integrating addiction medicine and hepatology remains challenging, and it is still an unmet goal in the field.^8^

Among the various strategies to address these challenges, peer-support or patient-support groups have emerged as a promising and underutilized resource.^9^^,^^10^ These groups provide a unique platform for individuals to share experiences, foster accountability, build trust, and offer emotional support that healthcare systems alone often cannot deliver.^11^^,^^12^ For patients with AUD and ALD, peer support can play a critical role in reducing stigma and promoting self-efficacy, both of which are essential for sustained recovery and adherence to treatments.^13^^,^^14^ Furthermore, peer groups often address psychosocial needs that are unmet in traditional clinical settings, such as providing a sense of belonging and community and reducing isolation and fear, which are particularly significant for patients with ALD who may experience heightened levels of shame and social withdrawal.^15^ By complementing medical care, peer support can help bridge gaps in the continuum of care, enhance patient engagement, and improve overall quality of life.^16^^,^^17^ This review aims to explore the current evidence, challenges, and future directions in leveraging patient-support groups to improve care for individuals with AUD and ALD, providing insights into their integration into existing care frameworks.

## THE ROLE AND IMPACT OF PEER AND PATIENT SUPPORT PROGRAMS

Various terms are used in the peer support landscape, and understanding these distinctions is critical. Patient support refers to programs or initiatives that provide individuals with chronic conditions or other health challenges with the resources, guidance, and community connections needed to navigate their care journeys.^16^^,^^18^ These programs bridge gaps in healthcare by addressing multidimensional needs that extend beyond clinical treatment, encompassing emotional, social, and practical support.^16^^,^^19^ A key component is assistance from individuals with lived experiences of a specific condition, which fosters understanding, empathy, and a unique connection often absent in traditional patient–provider interactions.^20^^,^^21^ The relatability and authenticity of peers can break down barriers to seeking help, reduce stigma, and create a sense of safety and trust.^22^ They often serve as role models by demonstrating resilience, offering hope, and providing practical guidance and emotional support.^23^

Peer support, a form of patient support, refers broadly to emotional, informational, and practical assistance from individuals with lived experience. Within this landscape, mutual aid groups such as AA or SMART Recovery are typically group-based, with members sharing similar recovery goals. Sponsors are more experienced peers in programs like AA who offer one-on-one mentorship, while peer navigators are trained peers integrated into clinical settings to help patients navigate healthcare systems and adhere to care plans. Peer support may be delivered one-on-one or in group settings, and while it shares some similarities with group psychotherapy, it is distinguished by its foundation in lived experience rather than professional training. Delivery formats include in-person and online groups, blogs, bulletin boards, and both real-time and asynchronous communication. These resources may or may not be professionally hosted or monitored, adding another layer of variability to user experience and outcomes.

Support groups have been effectively utilized across a variety of chronic medical conditions, including cancer, diabetes, heart failure, HIV/AIDS, and mental illness.^24^^–^^28^ For example, in oncology, peer support has been associated with improved coping skills and reduced psychological distress, while diabetes self-management groups have enhanced patient activation and treatment adherence.^29^^,^^30^ These models underscore the broad applicability and utility of peer support across diverse disease states, reinforcing its value in managing complex, chronic conditions such as AUD and ALD.

Patient support groups, a common and structured form of peer support, bring together individuals in a shared environment where they can discuss their experiences, share coping strategies, learn about their conditions, and offer mutual encouragement.^31^^,^^32^ These groups vary in format and structure, including in-person meetings, virtual communities, or hybrid models that combine face-to-face interaction with online engagement.^33^^,^^34^ This adaptability allows patient support groups to cater to diverse audiences, including individuals in rural areas, those with mobility challenges, or patients seeking culturally specific resources.^35^^,^^36^ As such, an additional added value of this approach is the feasibility of providing support in rural areas and other often underserved communities. The scope of these groups often extends beyond general discussions, focusing on specific subpopulations or tailored needs. Through their interactive nature, patient support groups effectively create a sense of belonging and understanding, which is particularly valuable for individuals who may feel isolated and alone due to their condition.^37^^–^^39^ Beyond emotional and social benefits, peer and patient support programs often address broader psychosocial aspects of health, complementing medical interventions in profound ways. They can help individuals navigate complex healthcare systems, adhere to treatment regimens, and build resilience in the face of adversity.^16^ Moreover, these programs empower participants by enhancing their knowledge and self-efficacy.^40^^,^^41^ In doing so, patient support initiatives not only improve individual outcomes but also contribute to better health equity by reaching populations that might otherwise lack access to comprehensive care.^40^^,^^41^

The success of peer and patient support programs can be attributed to several interconnected mechanisms that collectively enhance their impact on individuals’ well-being.^16^^,^^42^ At the core of these programs are shared experiences, a foundational element that fosters an immediate sense of connection and understanding among participants.^43^ By interacting with peers who have faced similar challenges, individuals often experience a deep validation of their struggles, helping to normalize emotions and reduce feelings of isolation and stigma.^44^ A key distinction between clinical and peer support relationships lies in the nature of personal disclosure. Clinical relationships are intentionally guided by professional boundaries and a degree of therapeutic distance; clinicians offer structured, objective guidance while maintaining professional opacity. In contrast, peer support relationships are built on mutual openness, personal storytelling, and direct access to one another’s lived experiences, sometimes spanning historical and real-time events in a person’s recovery journey. This transparency fosters trust and relatability in ways that differ fundamentally from clinician–patient interactions. Importantly, these bonds cannot, and arguably should not, be replicated in traditional clinical settings, as doing so could compromise the unique properties of a therapeutic alliance and the boundaries necessary for professional efficacy.^45^

Building on this foundation is emotional support, which provides participants with a safe, non-judgmental space to express their concerns, fears, and vulnerabilities.^44^^,^^45^ In this environment, individuals receive empathy, encouragement, and understanding from others who truly grasp the nuances of their experiences. The emotional validation and solidarity offered by peers can alleviate feelings of helplessness and provide a much-needed outlet for stress, further promoting mental and emotional well-being.^45^

Another important mechanism is accountability, which is uniquely facilitated by the peer dynamic.^46^ Through shared goals and mutual encouragement, participants are motivated to adhere to recovery plans, maintain lifestyle changes, and stay committed to treatment regimens.^46^ The accountability inherent in peer relationships helps sustain focus and determination. Equally important is knowledge sharing, as participants exchange practical advice, coping strategies, and valuable insights into navigating complex healthcare systems.^16^ This collective wisdom not only equips individuals with actionable tools to manage their conditions but also enhances their ability to advocate for themselves in healthcare settings.^17^^,^^47^

Role modeling further amplifies the impact of the peer and patient support programs.^48^ People with AUD often feel hopeless and believe that nothing can be done to overcome their alcohol problems. Observing peers who have successfully navigated similar challenges provides tangible examples of resilience, recovery, and hope.^49^ These role models serve as living proof that progress and healing are attainable, inspiring others to remain committed to their own journeys.^48^

Community building emerges as a transformative aspect of peer and patient support programs.^50^ These programs cultivate a network of solidarity and mutual upliftment, creating enduring connections that often extend beyond formal group settings.^21^ The sense of belonging to a community of like-minded individuals fosters a deep emotional resilience, as participants feel supported and valued within a collective.^21^ This ongoing support network can be a source of strength and encouragement during times of crisis or transition.

Finally, unlike clinical settings, where support is often framed through empathetic neutrality and unconditional positive regard, peer interactions can include forthright, candid, and even critical feedback. This brash honesty, though occasionally difficult to hear, can foster accountability and promote self-awareness in ways that resonate deeply with participants.

## CONCEPTUAL FRAMEWORK ON PATIENT-SUPPORT GROUPS TO IMPROVE CARE FOR INDIVIDUALS WITH AUD

Peer support in the treatment of AUD has deep historical roots, with Alcoholics Anonymous (AA) serving as one of the most well-known and enduring models of peer-based recovery.^51^^,^^52^ Founded in 1935, AA established the concept of mutual aid groups, where individuals struggling with alcohol problems could share their experiences and support one another in their recovery journey.^51^ AA’s success lies in its emphasis on shared experiences, the role of sponsors (more experienced members who guide newcomers), and the sense of fellowship that provides strength in numbers.^51^^–^^53^ Another peer support model, SMART Recovery (Self-Management and Recovery Training), takes a secular, evidence-based approach to addiction recovery.^54^^–^^56^ SMART Recovery focuses on cognitive-behavioral techniques and self-empowerment, with peer support integrated into its framework to help individuals achieve self-directed change.^54^^–^^56^ Compared with AA, SMART Recovery puts more emphasis on a more structured program with a focus on psychological resilience and skill-building.^54^^–^^56^ Both AA and SMART Recovery have contributed to the evolution of peer support in AUD care, each providing different avenues for mutual aid that cater to the diverse needs and preferences of those seeking help. Some peer support groups, such as AA and Celebrate Recovery,^57^ incorporate religious or spiritual elements that may enhance engagement for certain participants while serving as a barrier for those who prefer secular approaches. Providing patients with a variety of options, including secular models like SMART Recovery, helps ensure alignment with individual beliefs and preferences. In addition to these, other widely utilized peer support options include Al-Anon, which provides support specifically for families and friends affected by someone else’s alcohol use.^58^ These models, along with Women for Sobriety,^59^ tailored for individuals with AUD, and Sober Livers,^60^ which supports people affected by AUD, ALD, and those who have undergone liver transplantation due to alcohol-associated causes, as well as their care partners, illustrate the expanding role of peer support as a critical component of treatment and long-term recovery.

Numerous studies have examined the effectiveness of peer support in the treatment of AUD. Research consistently shows that peer support can significantly reduce alcohol consumption, with participants in peer-led groups like AA reporting higher rates of abstinence and reduced frequency of heavy drinking compared with those who do not engage in peer support programs.^52^ A meta-analysis of randomized controlled trials (RCTs) examining AA’s and clinically delivered 12-Step Facilitation interventions found that peer support participants had similar benefits to other treatments on all drinking-related outcomes except for continuous abstinence and remission, where AA’s and 12-Step Facilitation led to a higher likelihood of maintaining long-term sobriety compared with those receiving only traditional medical treatments such as counseling or pharmacotherapy.^61^ Moreover, peer support has been linked to improved mental health outcomes and common comorbidities among patients with AUD.^21^^,^^62^ Studies indicate that participants in peer-led recovery groups often report lower levels of anxiety, depression, and stress, likely due to the emotional support and sense of belonging provided by these groups.^21^ The shared experience of overcoming alcohol-associated struggles creates an empathetic environment where participants can discuss sensitive topics freely, thus enhancing psychological well-being. In addition, peer support offers an opportunity for role modeling and mentoring, where more experienced members can provide guidance and encouragement, boosting self-esteem and hope for recovery.^16^ The benefits of peer support are not limited to alcohol consumption alone; they extend to other areas of life as well. Peer support programs have been shown to enhance patients’ overall quality of life by improving social connectedness, reducing feelings of isolation, and fostering a sense of empowerment.^63^ There is a strong link between alcohol drinking and loneliness^64^ and specific to the focus of this review, an ecological momentary assessment study in people with AUD and ALD showed how features associated with craving were also significantly correlated with all moods, including loneliness and stress.^65^ Through consistent interaction with others who understand their experiences, individuals develop stronger coping mechanisms, improved self-efficacy, and greater resilience, all of which contribute to a more sustainable recovery process.

While peer support has demonstrated significant benefits in AUD care, several barriers to its widespread implementation and engagement exist.^66^ One major challenge is the variability in individuals’ readiness for peer support. Not all individuals with AUD are willing or ready to engage in peer support groups, especially in the early stages of treatment.^67^ The stigma surrounding alcohol use, coupled with feelings of shame or guilt, can prevent individuals from seeking help or opening up in group settings.^14^ In addition, some individuals may prefer individualized or professional care over group settings, which can limit the effectiveness of peer support for certain populations.^68^ Access to peer support programs can also be a barrier.^66^ This is particularly true for individuals living in rural or underserved areas where access to in-person support is limited.^69^ Moreover, transportation issues, work schedules, or caregiving responsibilities can impede regular participation in face-to-face peer support groups.^70^^–^^72^ While AA has a well-established online presence, virtual peer support options may still be underutilized due to user-level barriers such as limited access to technology or low digital literacy, rather than a lack of available resources.^34^^,^^73^ To overcome these barriers, innovative solutions such as virtual meetings, hybrid models, and community-based outreach are needed to increase accessibility and inclusivity.^72^ Another challenge to the success of peer support is the variability in group dynamics and facilitator quality.^74^ Not all peer support groups are equally structured or effective, and the success of these groups often depends on the leadership and commitment of group facilitators. A lack of trained facilitators or poor group cohesion can undermine the potential benefits of peer support. Facilitators for the successful implementation of peer support in AUD care include the increasing recognition of its importance within the broader healthcare system.^75^ As awareness of peer support’s effectiveness grows, more healthcare providers are incorporating it as part of comprehensive treatment plans for AUD. In addition, the expansion of digital platforms has facilitated greater engagement with remote or virtual peer support, increasing the reach of these programs. These technological advances, along with the development of culturally tailored programs, have enabled peer support to be more inclusive and accessible to a wider range of individuals, regardless of their background or location. Encouraging patient advocacy and involving family members in the process can also enhance engagement and reinforce the positive impact of peer support. In summary, peer support plays a vital role in AUD care by addressing emotional, psychological, and social needs that are critical to sustained recovery. In addition to community-based models, peer navigators embedded within medical settings are increasingly being utilized to bridge gaps in care, enhance treatment engagement, and support patients throughout their recovery journey. While challenges in accessibility and engagement persist, expanding and integrating peer support, including peer navigation, into both clinical and community settings holds promise for improving outcomes in individuals with AUD.

## CONCEPTUAL FRAMEWORK ON PATIENT-SUPPORT GROUPS TO IMPROVE CARE FOR INDIVIDUALS WITH ALD

The unique challenges faced by individuals with ALD make peer and patient support particularly relevant and impactful for these populations. AUD is often accompanied by stigma, feelings of shame, and social isolation, which can exacerbate mental health struggles and create significant barriers to seeking care.^76^ These psychological burdens can lead to a vicious cycle where the individual’s reluctance to seek help only deepens their condition. Similarly, ALD patients contend with a complex array of challenges, including the management of a chronic, progressive illness, and adherence to demanding medical regimens.^77^ The psychological toll of living with ALD, given its intrinsic etiological relationship with alcohol consumption, often includes feelings of guilt, shame, and hopelessness, which can further hinder recovery.^14^

Peer support plays a critical role in helping individuals with AUD and ALD navigate these multifaceted challenges.^78^ By offering a space where individuals can connect with others who have experienced similar struggles, peer support helps normalize their feelings, thus reducing stigma and fostering a sense of shared understanding. Moreover, emotional support from peers can be a powerful motivator, especially in the face of setbacks.^79^ Peers who have faced similar struggles provide encouragement and guidance, reinforcing the importance of perseverance in recovery and helping individuals stay committed to their recovery goals.^79^ For ALD patients, who often experience heightened levels of shame or guilt related to the progressive nature of their condition and its connection to alcohol use, peer support can be particularly transformative.^14^ Peer groups create a non-judgmental environment where individuals can reflect on their experiences, share coping strategies, and process difficult emotions in a supportive and empathetic space.^21^ This psychological healing is a vital complement to medical care, which can be impersonal and primarily focused on the physical aspects of underlying ALD. Peer support not only provides emotional validation but also promotes adherence to treatment plans, such as maintaining abstinence from alcohol and making necessary lifestyle changes like improving diet or exercising regularly.^9^ By creating a bridge between the emotional and practical aspects of recovery, peer support enables patients to better navigate the complexities of ALD treatment.

Patients with ALD face a range of unique challenges that make peer support particularly valuable. One of the most significant challenges is the stigma associated with alcohol use and liver disease.^14^ This stigma can lead to social isolation, reluctance to seek treatment, and a lack of self-compassion, all of which can exacerbate mental health issues like depression and anxiety.^14^^,^^80^ As a result, ALD patients may feel disconnected from both their healthcare providers and others, further compounding the emotional toll of the disease. In addition, many individuals with ALD struggle with complex comorbidities, including cardiovascular disease, diabetes, and mental health disorders, such as depression or AUD itself.^80^ These overlapping conditions can make treatment more difficult, requiring careful management of multiple health concerns. Furthermore, the progressive nature of liver disease means that many ALD patients eventually face advanced liver disease or cirrhosis, which may lead to the need for a liver transplant or result in significant limitations on daily functioning.^1^ These complications can be overwhelming and demoralizing, as patients often feel that their condition is beyond control, particularly when there is limited improvement despite adherence to treatment regimens. As a result, patients may struggle with hopelessness, which can make recovery efforts more difficult. These efforts may be further challenging by the medical complications of ALD itself, especially at its advanced stages, such as hepatic encephalopathy.

In this context, peer support programs provide essential emotional, informational, and social resources to help individuals navigate these complex challenges.^81^ Peer support offers ALD patients a sense of belonging and connection to others who understand their struggles.^82^ Sharing experiences with those who have faced similar challenges can help normalize feelings, reduce stigma, and provide reassurance during difficult times. Moreover, peer support can help patients with ALD manage the burden of their comorbidities by offering practical advice on lifestyle changes, coping mechanisms, and strategies for adhering to complex treatment regimens.^82^^,^^83^ In particular, peer support can assist in fostering motivation to engage in behavioral changes critical for liver health, such as abstinence from alcohol and the adoption of healthier diets and other healthy habits such as exercise.

The integration of peer support into ALD care has been increasingly recognized as a crucial aspect of managing the disease, especially as a complement to traditional medical interventions.^82^ Structured pretransplant care, including participation in addiction treatment, engagement with peer support programs such as AA, and sustained abstinence from alcohol, has been shown to improve posttransplant outcomes in patients with ALD. Transplant recipients with robust pretransplant recovery engagement, including peer support, had lower relapse rates.^84^ Programs that provide peer mentorship to transplant candidates offer a continuum of support from early abstinence through transplant evaluation and post-operative care. These initiatives exemplify how structured peer support can be seamlessly integrated into specialized medical pathways and highlight opportunities to replicate such models for all patients with ALD.

Peer mentors who have successfully navigated the challenges of ALD can provide hope and realistic expectations, empowering patients to continue with their recovery journey.^5^ Importantly, individuals who serve as peer mentors often experience enhanced recovery themselves. The act of guiding others fosters purpose, reinforces their own sobriety, and contributes to sustained engagement in recovery-oriented behaviors. For patients with ALD, peer navigators may be uniquely positioned to provide essential education and serve as a liaison between the patient and their healthcare team. Peer navigators are trained individuals who share a common health condition with the patients they support. They typically work under the supervision of a healthcare team and focus their efforts at the individual level. Peer navigators perform a variety of activities, including offering emotional, social, and practical support; educating patients about their health care needs; assisting with appointments and referrals; and advocating on behalf of patients. Drawing from their own lived experiences, peer navigators are well-equipped to help patients navigate complex healthcare systems, understand available resources and services, and advocate for themselves. Their shared experience enables them to identify relevant support systems and guide patients toward improved access to and quality of care. Table 1 demonstrates a summary of key peer support models relevant to AUD and ALD.

**TABLE 1:** Models of peer support relevant to AUD and ALD

Model	Description	Key Features	Setting/format	Potential benefits and relevance to ALD
Alcoholics Anonymous (AA)	Mutual aid group based on 12-step philosophy	Fellowship, sponsors, abstinence focus	In-person, hybrid, virtual	RCTs show improved abstinence and reduced drinking intensity in people with AUD^52^
SMART Recovery	CBT-based, secular mutual aid	Self-management, skill-building	Online, in-person	Potential improvements in mental and physical health and reducing alcohol use in people with AUD^56^
Peer Navigators	Trained peers integrated into clinical care	Appointments, linkage to care	Clinical or hybrid	Can assist in navigating the healthcare system and may improve treatment engagement
Sober Livers	Peer-led support for ALD/transplant patients and monthly educational sessions	ALD-specific, caregiver inclusive	Virtual and in-person	Provides educational sessions by medical experts on ALD and transplant, and peer support by persons with ALD and those who are waiting for or have undergone transplant
Celebrate Recovery	Christian 12-step peer program	Faith-based, abstinence focus	In-person, group-based	Valuable for faith-aligned patients
Al-Anon	Peer support for loved ones/friends/caregivers of individuals with AUD	Family-focused, emotional support	In-person/virtual	Provides support from caregiver peers, role modeling of coping, and more rewarding pursuits^85^
Online Forums & Apps	For example, Reddit, In the Rooms, Loosid	Anonymous, on-demand support	Digital, 24/7 access	Enhances accessibility

Abbreviations: ALD, alcohol-associated liver disease; AUD, alcohol use disorder; CBT, cognitive-behavioral therapy; RCTs, randomized controlled trials.

As healthcare delivery becomes increasingly digital, new models of peer support are emerging to meet the needs of ALD patients, especially those who face challenges related to accessibility, geography, and stigma.^86^ Telehealth interventions are becoming an integral part of liver disease care, particularly in remote or underserved areas where in-person meetings may not be feasible.^87^^–^^90^ Virtual peer support groups allow patients to connect with others from the comfort of their homes, breaking down geographical barriers and reducing the stigma associated with attending traditional in-person meetings.^91^ These virtual platforms can also offer a degree of anonymity, which may be especially beneficial for individuals who are hesitant to seek help in person due to the shame associated with their condition. Hybrid models, which combine both in-person and virtual peer support options, are gaining popularity as they offer flexibility and ensure that patients have access to support regardless of their location or schedule.^92^ This model also helps foster continuity of care by providing ongoing support throughout a patient’s treatment journey. For instance, some ALD care teams have implemented hybrid peer support programs that allow patients to participate in face-to-face meetings during hospital visits and access virtual sessions in between appointments. This approach ensures that patients stay connected to the support network even as their treatment progresses.

Culturally tailored peer support programs are another promising development in ALD care. Recognizing that ALD affects individuals from diverse backgrounds with varying cultural perspectives, these programs are designed to accommodate cultural differences in values, beliefs, and healthcare practices.^93^ For example, culturally adapted programs may involve support groups that address specific issues faced by minority groups or incorporate culturally relevant coping strategies. Tailoring peer support in this way can help ensure that all patients, regardless of their background, feel heard, understood, and respected. In addition, culturally sensitive peer support can help mitigate the stigma associated with alcohol use within certain cultural contexts, creating a safe space for individuals to discuss their condition and recovery.^94^

## INNOVATIONS AND FUTURE DIRECTIONS

The integration of technology into peer support systems is revolutionizing access and effectiveness, making these resources more widely available and adaptable to modern lifestyles. Smartphone apps, telemedicine, and virtual platforms are playing increasingly significant roles in connecting individuals who might otherwise be unable to participate in traditional peer support groups due to geographical, logistical, or personal barriers.^95^^,^^96^ Mobile apps, in particular, are providing new frontiers for peer support. These platforms often incorporate features such as group chats, one-on-one messaging, forums, and even real-time emotional support. Some apps are designed to integrate with wearable technology, enabling users to track their physical and mental health progress while engaging with peer communities for encouragement and accountability. Apps tailored to AUD and ALD often include tools such as sobriety trackers, daily check-ins, and motivational content, creating a holistic environment for recovery.^97^^–^^99^ Furthermore, it is clear that artificial intelligence (AI)-driven approaches will play a wider and wider role in medicine, including in the diagnosis, prognosis and treatment of patients with AUD and ALD (for review, see reference^100^), therefore it is conceivable that AI-based approaches may improve, facilitate and help the integration of peer support in the context of AUD and ALD.

Telemedicine has expanded the reach of peer support by enabling virtual group meetings and individual sessions facilitated by healthcare providers or peer mentors. These virtual platforms are particularly impactful for individuals in rural or underserved areas who may lack access to in-person groups. Moreover, telehealth reduces stigma by allowing individuals to participate from the privacy of their own homes, which can encourage those hesitant to seek help in public settings.

As peer support evolves, there is a growing emphasis on tailoring these programs to meet the diverse needs of individuals based on sex, age, cultural background, or disease stage. Personalized support groups acknowledge that recovery is not a one-size-fits-all process and that specific subgroups may face unique challenges requiring specialized attention. For example, sex-specific support groups address the distinct experiences of men and women in AUD and ALD recovery.^101^^,^^102^ Women, who may experience greater stigma or specific barriers to seeking help, often benefit from a supportive environment tailored to their unique concerns, such as balancing caregiving responsibilities with recovery efforts.^101^^,^^103^ Peer support programs designed to respect cultural values, beliefs, and traditions can foster greater engagement and trust among participants. For instance, culturally specific groups may integrate faith-based elements, language preferences, or traditional coping strategies that resonate more deeply with participants from certain backgrounds. Disease stage-specific groups are also valuable, as individuals in early stages of ALD may have different needs and concerns than those facing advanced liver disease or awaiting a liver transplant.^104^

The future of peer support lies in its seamless integration with medical and psychological care, creating a comprehensive, multidisciplinary approach to AUD and ALD management. Collaborations between peer support programs and healthcare teams enable a more coordinated and patient-centered model of care, addressing both the medical and psychosocial dimensions of these conditions. Peer support can complement healthcare services by providing emotional and practical guidance that healthcare providers may not have the expertise, time, or resources to offer during routine appointments. For example, peer mentors can assist patients with ALD in better understanding complex medical information, including the risks and benefits of various treatment options and the importance of adhering to prescribed medications. Peer support can also reinforce lifestyle changes recommended by healthcare providers, such as maintaining abstinence, improving nutrition, or incorporating exercise. Healthcare teams can benefit from peer support by gaining insights into patients’ experiences and challenges, which can inform more personalized treatment plans. Collaborative models may involve training peer mentors to recognize red flags for medical or psychological issues that require professional intervention, ensuring that patients receive timely and appropriate care. In some settings, peer mentors are integrated directly into healthcare teams, attending appointments with patients or participating in multidisciplinary case discussions. In addition, the inclusion of mental health professionals such as psychologists, psychiatrists, and clinicians specializing in the treatment of AUD in peer support collaborations ensures that patients with co-occurring mental health disorders receive comprehensive care.^105^

**FIGURE 1:**
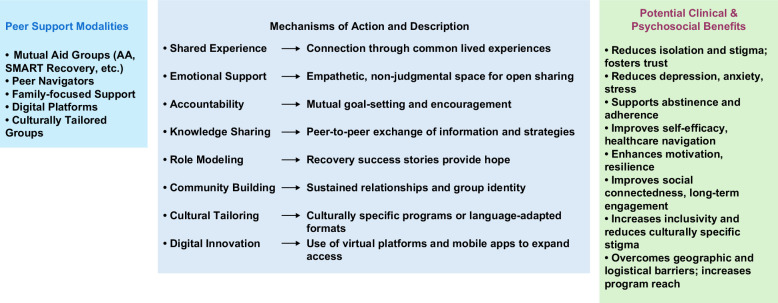
Conceptual framework illustrating pathways by which peer support interventions influence outcomes in alcohol-associated liver disease. Peer support modalities (eg, mutual aid groups, peer navigators, condition-specific groups, family-focused support, digital platforms, and culturally tailored groups) engage participants through mechanisms of action such as shared experience, emotional support, accountability, knowledge sharing, role modeling, and community building. These mechanisms contribute to potentially improved clinical outcomes and psychosocial outcomes.

Finally, it will be important to shed light on the mechanisms of how peer support works in order to develop better and more personalized approaches. This will also help fortifying the importance of making peer support a central core of the management of patients with AUD, including and especially those with ALD. These future directions could include both human studies aimed at studying peer support via controlled human studies, and patient-driven reverse translational preclinical studies aimed at shedding light on biological and molecular mechanisms. Indeed, recent exciting neuroscience work in rat models of addiction shows the key role and the molecular signatures of social reward in reducing drug-seeking and taking (for reviews, see references^106^^,^^107^). In line with these important future directions, it is also important to keep in mind the growing evidence on the need for patient-driven input (via, eg, community advisory boards) in the design, implementation, and execution of clinical research. Figure 1 illustrates the conceptual framework by which peer support interventions engage patients through mechanisms such as shared experience, emotional support, accountability, knowledge sharing, role modeling, and community building, ultimately leading to potentially improved clinical and psychosocial outcomes in individuals with AUD and ALD.

## CHALLENGES AND LIMITATIONS

While peer support has emerged as a promising component of care for individuals with AUD and ALD, realizing its full potential requires overcoming significant challenges and limitations. These obstacles encompass implementation barriers, research gaps, and ethical and logistical concerns, all of which highlight the need for strategic solutions and sustained evaluation efforts. As peer support becomes more integrated into healthcare delivery, it is essential to define clear boundaries and roles to prevent mission creep. While peer mentors play a vital supportive role, clinical decision-making must remain within the scope of licensed professionals. Ensuring legal clarity and establishing protocols for supervision and communication between peers and providers are critical for maintaining patient safety and trust. Many individuals with AUD and ALD face stigma or denial about their conditions, which can deter them from joining or staying engaged in peer support programs. Logistical challenges such as transportation, scheduling conflicts, and caregiver responsibilities further hinder consistent participation, while fluctuating motivation and periods of relapse complicate long-term engagement. Another major barrier is funding and sustainability. Unlike medical interventions, peer support is often viewed as complementary or supplementary, making it challenging to secure consistent funding. Programs that rely on volunteers may struggle with maintaining adequate training, supervision, and support for peer mentors, which is essential for program sustainability and effectiveness. In addition, integrating peer support into existing healthcare systems presents obstacles. Resistance from healthcare providers unfamiliar with or skeptical of peer support’s benefits can impede collaboration. Establishing clear roles and communication pathways between peer mentors and medical teams requires considerable effort and coordination. Research gaps also hinder progress, particularly the limited availability of high-quality evidence specific to ALD populations. Unlike AUD, ALD presents unique complexities, such as its progressive nature and frequent comorbidities, necessitating tailored peer support interventions. Further studies are needed to evaluate the impact of peer support on clinical outcomes in ALD, such as treatment adherence, nutritional improvements, and hospitalization rates. In addition, the lack of standardized metrics for measuring the success of peer support programs poses a challenge. Existing evaluations often rely on subjective outcomes like sobriety or quality of life, which may not fully capture program effectiveness or areas for improvement. Rigorous methodologies and validated measures are needed to build a robust evidence base. Ethical and logistical issues further complicate implementation. Privacy and confidentiality concerns arise as participants share personal experiences, particularly in virtual or hybrid settings where data security may be vulnerable. Clear guidelines and safeguards are essential to maintain trust and protect sensitive information. In addition, a frequent concern in rural or tightly knit communities is the potential loss of anonymity in local, in-person peer support meetings. Social overlap in these small communities can lead to fear of judgment, stigma, or unintended disclosure of personal information. This lack of perceived confidentiality can be a significant deterrent to participation in traditional peer support groups, highlighting the need for tailored approaches that address these unique challenges.

## CONCLUSIONS

Peer support is becoming an increasingly important part of care for people with AUD and ALD. It offers benefits that go beyond traditional medical or psychological treatments. By sharing their own experiences, peer mentors provide emotional support, reduce stigma, offer practical advice, and help create a sense of community. They also help patients stay motivated and deal with treatment challenges.

Peer support can be delivered in many ways, online, in culturally tailored groups, or through hybrid formats, making it more accessible, especially for people in rural or underserved areas. However, challenges remain. These include finding and retaining peer mentors, securing funding, and ensuring long-term program success. There is also a lack of research focused specifically on peer support for people with ALD. To make peer support a regular part of care, researchers, healthcare providers, and policymakers need to work together. This means investing in mentor training, integrating programs with medical care, and updating policies to support these services. With the right support, peer programs can improve treatment, support lasting recovery, and enhance the lives of people living with AUD and ALD.
